# Spinal Anesthesia in a Respiratory-Compromised Patient With Fabry Disease Undergoing Umbilical Hernia Repair: A Case Report

**DOI:** 10.7759/cureus.103898

**Published:** 2026-02-19

**Authors:** Paraskevi Mavridou, Christos Exarchos, Panagiota Panagiotou, Pinelopi Kitsakou, Chrysoula Antigoni Koutsi

**Affiliations:** 1 Department of Anesthesiology, General Hospital of Ioannina "G. Hatzikosta", Ioannina, GRC

**Keywords:** anesthesia spinal, fabry disease, obesity, obstructive lung disease, perioperative management, umbilical hernia repair

## Abstract

Fabry disease (FD) is a rare X-linked lysosomal storage disorder associated with multisystemic involvement, making anesthetic management challenging due to potential cardiomyopathy, renal insufficiency, and airway difficulties. We present the case of a 42-year-old female patient with FD and significant respiratory comorbidities, including active smoking and moderate-to-severe airflow obstruction (FEV1: 50% predicted), alongside a body mass index (BMI) of 32.3 kg/m², presenting for umbilical hernia repair. Given the high risk of perioperative respiratory complications, spinal anesthesia was selected. A subarachnoid block performed at L2-L3 achieved a T6 sensory level. The patient maintained hemodynamic stability without vasopressor support and experienced no respiratory or neurological complications. This case contributes to the scarce literature on neuraxial anesthesia in FD, demonstrating that spinal anesthesia can be safely extended to abdominal wall procedures requiring higher dermatomal levels (T6), provided that hemodynamic monitoring is rigorous.

## Introduction

Fabry disease (FD) is a rare X-linked progressive lysosomal storage disorder caused by alpha-galactosidase A deficiency, with an estimated incidence ranging from 1 in 40,000 to 1 in 117,000 live births. This enzymatic defect leads to the systemic accumulation of globotriaosylceramide (Gb3) in the lysosomes of various tissues, particularly the vascular endothelium, heart, kidneys, and autonomic nervous system [1]. Crucially for the anesthesiologist, this cellular pathology directly translates into organ-specific risks: glycosphingolipid deposition stiffens the myocardium, leading to diastolic dysfunction, infiltrates conduction pathways, causing arrhythmias, and accumulates in soft tissues, narrowing the upper airway. The standard of care involves life-long enzyme replacement therapy (ERT) to reduce substrate accumulation and slow disease progression [2].

Anesthetic management of patients with FD presents significant challenges due to the disease's multisystemic nature. Patients often present with cardiomyopathy (left ventricular hypertrophy, arrhythmias), renal impairment, cerebrovascular disease, potential difficult airway from glycosphingolipid deposition, and autonomic dysfunction [3-5]. While general anesthesia (GA) is commonly described, it carries inherent risks of myocardial depression and respiratory complications, particularly in patients with pre-existing pulmonary pathology [6].

We report the successful spinal anesthetic management of a patient with FD, significant pulmonary comorbidity, and obesity who underwent umbilical hernia repair, thereby supporting the feasibility of achieving a T6 sensory level for abdominal wall surgery under strict hemodynamic and respiratory monitoring.

## Case presentation

A 42-year-old female patient with a weight of 88 kg and a height of 165 cm (BMI 32.3 kg/m ², obesity class I) presented for elective repair of a symptomatic umbilical hernia. She had a confirmed diagnosis of FD, inherited from her father, on biweekly enzyme replacement therapy (agalsidase beta). Clinically, she reported chronic acroparesthesia managed with pharmacotherapy and limited exercise tolerance (NYHA Class II) primarily due to respiratory symptoms, but denied a history of stroke or transient ischemic attacks.

Her medical background was complex and included arterial hypertension treated with irbesartan/hydrochlorothiazide and amlodipine, cardiac arrhythmias managed with propafenone, and heterozygous beta-thalassemia. Of particular concern was her respiratory status: the patient suffered from asthma and was an active smoker (10 pack-years). She reported a severe episode of bronchospasm 20 days prior to admission requiring systemic corticosteroids and intensification of her inhaler therapy (beclomethasone/formoterol). Notably, the patient reported "delayed emergence" and severe cervical pain following a previous GA, creating significant anxiety regarding future sedation.

Clinical examination and diagnostic tests

Preoperative physical examination revealed audible inspiratory and expiratory wheezing upon lung auscultation, without clinical signs of acute infection. Airway assessment was Mallampati Class II. Screening for obstructive sleep apnea (OSA) revealed a STOP-BANG score of 1 (positive only for hypertension), indicating low risk. Laboratory investigations showed microcytic hypochromic anemia (hemoglobin 10.7 g/dL), consistent with her thalassemia trait. Renal function was preserved but borderline (Serum Creatinine 1.00 mg/dL). Electrolytes and coagulation profile were within normal limits (Table 1).

**Table 1:** Preoperative laboratory results ALP: alkaline phosphatase; ALT: alanine aminotransferase; aPTT: activated partial thromboplastin time; AST: aspartate aminotransferase; CK: creatine kinase; γGT: gamma-glutamyl transferase; Hb: hemoglobin; Hct: hematocrit; HDL: high-density lipoprotein; INR: international normalized ratio; LDH: lactate dehydrogenase; MCH: mean corpuscular hemoglobin; MCV: mean corpuscular volume; PLT: platelets; WBC: white blood cells

Parameter	Preoperative value	Reference range
RBC	4.44 M/μL	3.8 – 6 M/μL
Hb	10.7 g/dL	11.8 - 17.8 g/dL
Hct	34.1 %	36 - 52 %
MCV	76.8 fL	80 - 96 fL
MCH	24.1 pg	26 - 32 pg
WBC	6.91 K/μL	4.0 - 11.0 K/μL
PLT	297 K/μL	140 - 450 K/μL
INR	0.96	1 – 1.3
aPTT	33.2 sec	25 – 38 sec
Glucose	102 mg/dL	70 - 115 mg/dL
Urea	37 mg/dL	10 - 50 mg/dL
Creatinine	1.00 mg/dL	0.5 - 1.1 mg/dL
Total Protein	6.7 g/dL	6.2 - 8.4 g/dL
Albumin	4.4 g/dL	3.5 - 5.1 g/dL
Potassium (K+)	4.4 mmol/L	3.5 - 5.1 mmol/L
Sodium (Na+)	139 mmol/L	136 - 146 mmol/L
Magnesium (Mg++)	1.85 mEq/L	1.3 - 2.1 mEq/L
Calcium (Ca++)	9.1 mg/dL	8.2 - 10.5 mg/dL
AST	23 U/L	5 - 33 U/L
ALT	24 U/L	5 - 32 U/L
γGT	27 U/L	5 - 31 U/L
ALP	75 IU/L	35 - 125 IU/L
LDH	242 IU/L	120 - 230 IU/L
CK	162 IU/L	0 - 220 IU/L

Respiratory and cardiac assessment

**Figure 1:**
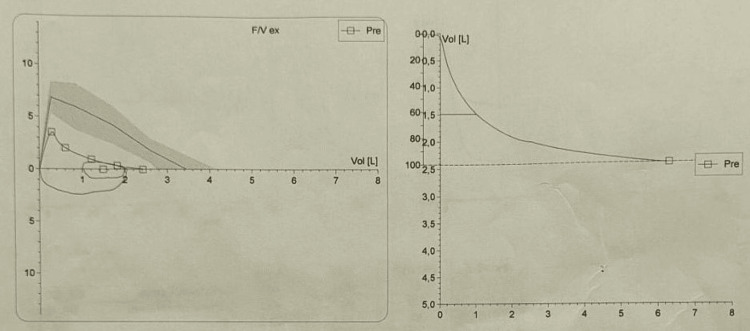
Preoperative spirometry flow-volume loop The flow-volume loop demonstrates a mixed ventilatory defect. The concave shape of the expiratory limb indicates airflow obstruction, while the reduced volume suggests a restrictive component.

Spirometry demonstrated a mixed ventilatory defect (Figure 1).

The FEV1 was 1.49 L (50% of predicted), and the forced vital capacity (FVC) was 2.42 L (70% of predicted). The FEV1/FVC ratio was 61%, confirming moderate-to-severe airflow obstruction (Table 2). 

**Table 2:** Preoperative pulmonary function tests % (A1/P): actual value expressed as a percentage of the predicted value; Act1: actual (measured) value; FEV 1: forced expiratory volume in 1 second; FVC: forced vital capacity; MEF: maximal expiratory flow; MMEF: maximal mid-expiratory flow; PEF: peak expiratory flow; Pred: predicted value

Parameter (Units)	Pred	Act1	% (A1/P)
FEV 1 (L)	2.99	1.49	50
FVC (L)	3.46	2.42	70
FEV 1 % FVC (%)	81	61	76
PEF (L/s)	6.87	3.54	52
MEF 25 (L/s)	1.82	0.37	20
MEF 50 (L/s)	4.23	0.95	22
MEF 75 (L/s)	5.96	2.05	34
MMEF 75/25 (L/s)	3.59	0.82	23

**Figure 2:**
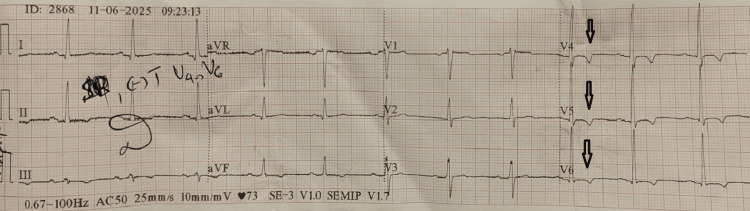
Preoperative 12-lead electrocardiogram (ECG) The tracing demonstrates normal sinus rhythm with distinct T-wave inversions in the precordial leads V4–V6. These repolarization abnormalities are suggestive of early Fabry cardiomyopathy, often preceding the development of overt left ventricular hypertrophy on echocardiography.

Transthoracic echocardiography showed a preserved left ventricular ejection fraction (LVEF) of 65%, without hypertrophy. However, the preoperative 12-lead electrocardiogram revealed sinus rhythm with T-wave inversions in precordial leads V4-V6 (Figure 2), suggestive of early FD cardiomyopathy preceding structural remodeling [7].

Anesthetic management

A multidisciplinary risk assessment identified that the combination of severe active bronchial obstruction, recent bronchospasm, obesity, and the theoretical risk of FD-related airway infiltration made GA high-risk for perioperative respiratory adverse events. Consequently, spinal anesthesia was selected.

Standard monitoring was applied (five‑lead ECG, non‑invasive blood pressure, and pulse oximetry), and airway equipment and vasopressors were prepared. The patient received prophylaxis for nausea (ondansetron 4 mg, omeprazole 40 mg). Under aseptic conditions, the subarachnoid space was accessed at the L2-L3 interspace using a 27-gauge atraumatic pencil-point spinal needle. A mixture of isobaric ropivacaine 0.75% (2.5 mL; 18.75 mg) and intrathecal fentanyl (15 µg) was administered. Immediately after injection, the patient was placed supine in a neutral position to avoid uncontrolled cephalad spread. Sensory level was assessed bilaterally with cold sensation every two to three minutes, and motor block was evaluated clinically. A T6 sensory level was achieved within 10 minutes. After block establishment, respiratory tolerance was reassessed (ability to speak full sentences, absence of dyspnea, respiratory rate, and SpO₂), and the patient remained comfortable without subjective or objective signs of respiratory compromise.

Surgery course and outcome

The surgery lasted 90 minutes. Intraoperatively, the patient remained hemodynamically stable, with mean arterial pressure (MAP) maintained between 85-100 mmHg and heart rate (HR) between 60 and 75 beats/min, with the administration of 1.5 L balanced crystalloids. A predefined threshold for hemodynamic intervention (MAP <80 mmHg or symptomatic hypotension) was used; vasopressors were immediately available but not required. Oxygen saturation remained >98% with supplemental oxygen 3 L/min via nasal cannula. Postoperatively, she had a smooth recovery with full regression of the motor block within three hours and was discharged the following day without complications.

## Discussion

Literature regarding the use of regional anesthesia in FD is scarce. Published reports are currently limited to obstetric procedures (cesarean section) [8] and surgeries involving the lower extremities [9]. This case highlights critical considerations in the anesthetic management of high-risk FD patients. First and foremost, a close interdisciplinary approach is essential, as multiple organ systems may be involved. Preoperatively, all disease-related comorbidities should be reassessed, particularly those affecting organs that may be compromised by the condition, including the heart, lungs, kidneys, and the autonomic nervous system. Such a multidisciplinary strategy is consistent with the "Center of Excellence" model described by Lee et al. [10].

Our rationale for choosing neuraxial anesthesia was primarily driven by respiratory safety. Our patient presented a constellation of risk factors: severe airflow obstruction, airway hyperreactivity, and obesity. GA is a potent trigger for bronchospasm in such patients. Respiratory involvement is increasingly recognized in FD; in the National Danish Fabry cohort, abnormal lung function variables were present in 28% of patients (20% obstructive, 8% restrictive), with obstructive limitation also observed in never smokers and worsening with disease severity [11]. Furthermore, airway management in FD can be notoriously difficult due to the accumulation of glycosphingolipids in the soft tissues of the oropharynx, leading to macroglossia and laryngeal narrowing [3,4]. The combination of obesity and potential FD-specific airway changes creates a hazardous scenario for tracheal intubation. By choosing spinal anesthesia, we bypassed airway instrumentation and avoided respiratory depressants, mitigating risks such as unexpected post-extubation respiratory failure described by Krüger et al., while the patient’s persistent preference was also taken into consideration [6].

Regarding hemodynamic management, a major theoretical concern with high-level spinal anesthesia (T6) in FD is the unpredictability of the autonomic nervous system. Autonomic neuropathy is common in FD and theoretically increases the risk of profound hypotension following sympathetic blockade. This underscores the need for vigilant monitoring and detailed preoperative cardiac assessment, essential in FD and multisystem genetic diseases with pre-excitation, to identify arrhythmia risk and occult cardiac involvement [12]. While a titratable technique, such as continuous epidural or combined spinal-epidural anesthesia, could theoretically offer more gradual hemodynamic control, we opted for single-shot spinal anesthesia to ensure a dense, reliable sensory block for surgery, managing hemodynamic stability through volume loading. Our patient maintained remarkable stability, which aligns with the two currently available reports of spinal anesthesia in FD by Kim et al. [9], who described successful spinal anesthesia for hallux valgus surgery, and Politt and Gaik [8], who reported an uncomplicated Cesarean section without major hemodynamic instability or neurological sequelae. Together with our experience, these reports suggest that neuraxial techniques can be feasible in FD across different surgical settings when individual cardiovascular risk is rigorously assessed. We attribute this stability to careful local anesthetic dosing, neutral patient positioning, and adequate volume loading utilizing balanced crystalloids and predefined MAP targets. The absence of significant hypotension suggests that higher thoracic levels may be tolerated in selected patients with FD without advanced cardiomyopathy, though this should not be extrapolated to all FD phenotypes.

Finally, neuraxial anesthesia provided dense intraoperative anesthesia and early postoperative analgesia, reducing the need for systemic opioids and sedatives. This is particularly beneficial not only in the setting of severe airflow obstruction but also for patients with FD at risk of progressive nephropathy, making the avoidance of nephrotoxic agents, such as non-steroidal anti-inflammatory drugs (NSAIDs), a priority [13]. In this context, effective neuraxial analgesia minimized the reliance on these agents, aligning with renal preservation strategies.

## Conclusions

This case expands the application of neuraxial anesthesia in Fabry disease to include abdominal wall surgery, demonstrating its feasibility even when a high dermatomal block (T6) is required. Spinal anesthesia offers a valuable alternative for patients with FD, concurrent respiratory compromise, and obesity by completely bypassing the risks of difficult airway management and respiratory depression associated with general anesthesia. Furthermore, our findings challenge the theoretical concern of profound autonomic instability, showing that hemodynamic control is achievable with rigorous monitoring and volume optimization. Ultimately, a tailored, multidisciplinary approach that prioritizes organ protection, specifically respiratory and renal preservation, is essential for improving perioperative outcomes in this high-risk population.
